# Cortical Bone Loss in Barcelona Across Time (1st Century CE–18th Century CE) and Its Potential Relationship With Linear Enamel Hypoplasia

**DOI:** 10.1002/ajpa.70241

**Published:** 2026-04-03

**Authors:** Antony Cevallos, Alexandre Tarragó, Carme Rissech

**Affiliations:** ^1^ Unitat d'Anatomia i Embriologia Humana, Facultat de Medicina i Ciències de La Salut Universitat Rovira i Virgili Reus Spain; ^2^ Acadèmia de Ciències Veterinàries de Catalunya Barcelona Spain

**Keywords:** Barcelona, cortical bone loss, diachronic study, linear enamel hypoplasia, paleopathology, pèrdua d'os cortical, estudi diacrònic, hipoplàsia lineal de l'esmalt, paleopatologia, Barcelona

## Abstract

**Objectives:**

The purpose of this study is to conduct a diachronic analysis of cortical bone loss in Barcelona, spanning from the 1st to 18th century CE, assessing the relationship between cortical bone loss and early‐life stressors, as indicated by linear enamel hypoplasia (LEH) across Roman, Late Antiquity, Medieval, and Modern periods and examines.

**Materials and Methods:**

A total of 226 adult individuals (117 males and 109 females) were analyzed. Radiogrammetry of the second metacarpal was used to calculate the metacarpal cortical index (MCI). A subsample of 153 individuals with anterior dentition was used to assess the association between MCI and LEH. Statistical analyses evaluated differences in MCI by sex, age group, historical period, and LEH. Depending on the results, parametric (Student's *t*‐test or one‐way ANOVA) or non‐parametric tests (Mann–Whitney *U* or Kruskal–Wallis tests) were applied.

**Results:**

No statistically significant differences in MCI were observed by sex, age group, or historical period. In contrast, individuals exhibiting LEH showed significantly lower MCI values compared to those without LEH (*p* < 0.05).

**Conclusions:**

The findings indicate that early‐life stressors may play a more important role in adult bone health than broad demographic variables, suggesting that early‐life stressors may negatively affect bone health in adulthood, supporting early‐life programming theory. This study provides the first evidence in Spain of cortical bone loss as assessed through radiogrammetry and is the first to explore the relationship between MCI and LEH, which can establish a foundation for future paleopathological research and comparative studies in Europe.

## Highlights

- First long‐term diachronic study of cortical bone loss in Barcelona (1st–18th century CE).
- Analyzed 226 adult skeletons using second metacarpal radiogrammetry to assess the metacarpal cortical index (MCI).
- A subsample of 153 individuals with observable mandibular dentition was used to explore the potential relationship between cortical bone loss and linear enamel hypoplasia (LEH).
- No significant differences in MCI were observed by sex, age, or historical period; however, individuals who exhibited LEH had significantly lower MCI than those who did not.
- The presence of LEH shows a potential relationship with lower MCI, linking stressors in early life to adult bone health, supporting early life programming theory.

## RESUM

**Objectius:**

L'objectiu d'aquest estudi és dur a terme una anàlisi diacrònica de la pèrdua d'os cortical a Barcelona, des del segle I fins al segle XVIII dC, tot avaluant la relació entre aquesta pèrdua òssia i els estressors patits durant la primera infància, tal com s'indica mitjançant la hipoplàsia lineal de l'esmalt (LEH), al llarg dels períodes romà, de l'antiguitat tardana, medieval i modern.

**Materials I mètodes:**

Es van analitzar un total de 226 individus adults, 117 homes i 109 dones. Es va aplicar la radiogrametria del segon metacarpià per calcular l'índex cortical metacarpià (MCI). Es va utilitzar una submostra de 153 individus amb dentició anterior conservada per avaluar l'associació entre l'MCI i la presència de LEH. Les anàlisis estadístiques van avaluar les diferències en el MCI segons el sexe, el grup d'edat, el període històric i la presència del LEH. En funció dels resultats, es van aplicar proves paramètriques, com la prova t de Student o l'ANOVA d'una via, o bé proves no paramètriques, com les proves U de Mann–Whitney o de Kruskal‐Wallis.

**Resultats:**

No es van observar diferències estadísticament significatives en el MCI segons el sexe, el grup d'edat o el període històric. En canvi, els individus que presentaven LEH mostraven valors de MCI significativament més baixos en comparació amb aquells que no en presentaven (*p* < 0.05).

**Conclusions:**

Els resultats indiquen que els factors d'estrès durant les primeres etapes de la vida poden tenir un paper més rellevant en la salut òssia adulta que les variables demogràfiques generals. Això suggereix que l'estrès infantil pot afectar negativament la salut òssia en l'edat adulta, donant suport a la teoria de la programació en els primers anys de vida. Aquest estudi aporta la primera evidència a Espanya de la pèrdua d'os cortical avaluada mitjançant radiogrametria i és el primer a explorar la relació entre el MCI i el LEH, establint així una base per a futures investigacions.

## 1 Introduction

Osteoporosis is a systemic metabolic disease characterized by decreased bone mineral density (BMD) and the deterioration of bone microarchitecture, leading to increased bone fragility and a heightened risk of fractures (Smith and Wordsworth 2005). Often referred to as a “silent epidemic,” it progresses asymptomatically and typically remains undiagnosed until a fracture occurs, which frequently happens years after menopause in women (Aibar‐Almazán et al. 2022) and in men older than 70 years (Watts et al. 2012). Although most prevalent among postmenopausal women, osteoporosis affects individuals across all demographics, including men, children, adolescents, and premenopausal women (Grover and Bachrach 2017; Harvey et al. 2019). Osteoporosis is a multifactorial disorder, resulting from the interaction of genetic, environmental, and cultural factors (Peacock et al. 2002), but from early adulthood there is always a decrease in bone mass relative to aging. The low bone density achieved in later life is strongly related to the amount of peak bone mass attained by young adulthood (Riggs 1991; Specker et al. 2010), which may explain the individual, sexual, and population differences in its incidence. In recent years, there has been a dramatic rise in the prevalence of bone loss and osteoporosis within increasingly senescent Western populations (Naso et al. 2025). Although this condition has generated a great deal of contemporary interest, its epidemiology in contemporary and past populations remains unclear (Curate 2014; Kruger and Wolber 2016; Zhang et al. 2020). Clinical research into osteoporosis is ongoing; however, it is only by turning to the archaeological record that we can track the prevalence and impact of this disease in past populations. Specifically, skeletal studies of ancient populations provide direct evidence of diseases and permit elucidation of the environmental and social context of those individuals affected (e.g., Rissech et al. 2013; Cevallos et al. 2024). By using archaeological evidence, we can understand the impact of changes in lifestyle (e.g., physical activity, nutrition, demographics, and socio‐economic context) beyond the parameters of modern clinical conditions, where patients tend to represent a narrower range of lifestyles. Skeletal studies of ancient populations can also provide a unique insight into the pathogenesis of bone loss and osteoporosis, thus contributing to medical history (e.g., Beauchesne and Agarwal 2014, 2017; Brødholt et al. 2021).

To understand the evolution of any pathology over time and its relationship with sex, age, lifestyle, and temporal transitions, it is necessary to focus analysis on a specific geographical area, evaluating different populations through time. In this way, the influence of geographical factors is reduced and the interpretation of the results simplified. It is known that paleopathological studies offer valuable direct evidence of disease, helping to clarify causes and trace disease progression, and that archaeological research on bone loss and osteoporosis has grown substantially in recent years (e.g., Spinek et al. 2016; Curate et al. 2019; Lorkiewicz et al. 2019). However, despite these advances, many important aspects of bone loss and osteoporosis remain incompletely understood. What is necessary is a spatially restricted, but temporally broad and complete (multivariate) overview of how the nature and frequency of this disease have changed through time, allowing new insights into the development of this disease and its underlying causal factors. In addition, these studies are more informative when it comes to the interpretation of bone loss on a broader time scale in a regional context because of the continuity in methodology and sample selection process (e.g., Lorkiewicz et al. 2019; Brødholt et al. 2021). Having control over the geographical factors of the individuals where they were alive and the factors of preservation and data collection will reduce the noise associated with bone density studies and improve the reliability of direct comparisons between two or more skeletal assemblages.

Understanding patterns of cortical bone loss is essential for assessing bone health and serves as a valuable diagnostic tool for identifying conditions such as osteoporosis (S. Mays 1996; Guerri et al. 2018). A previous anthropological study from Ives and Brickley (2005) found that the cortical index of the second metacarpal correlates well with BMD in other elements of the skeleton, in particular with the vertebrae and the distal radius. In both clinical and research settings, measurements of cortical bone are used to monitor and evaluate this bone loss over time (Ashok et al. 2018; O'Mara et al. 2024). In bioarchaeology, interest in bone loss and osteoporosis has grown as researchers seek to uncover historical trends and the prevalence of these conditions (Agarwal 2018). Within paleopathology, such studies provide insights into age‐ and sex‐related changes in cortical bone among past populations, offering valuable information about how lifestyle factors may have influenced skeletal health (Curate 2014; van Spelde et al. 2021). Radiogrammetry, introduced in the 1960s, is a non‐invasive technique that uses standard radiographs, typically of the metacarpals, to assess cortical bone thickness and detect age‐related skeletal changes (Barnett and Nordin 1960; Virtamä and Mähönen 1960). While plain radiographs can evaluate cortical thickness, bone translucency, and fractures, they are not sufficient on their own to provide a definitive diagnosis of conditions such as osteoporosis (Anil et al. 2010; Link 2016). However, radiogrammetry remains a valid method for assessing cortical bone loss. It continues to be a useful tool in clinical settings, particularly in environments where access to advanced imaging technologies is limited (O'Mara et al. 2024). Furthermore, it has shown potential in predicting future osteoporotic fracture risk, especially among women (Ashok et al. 2018).

Traditionally focusing on the second metacarpal, radiogrammetry has proven to be a cost‐effective and non‐destructive method for detecting bone loss in European archaeological populations (e.g., S. Mays 2000; Ives and Brickley 2005; Glencross and Agarwal 2011). This technique has also been successfully adapted for use on fragmentary skeletal remains, offering an efficient method for assessing cortical bone loss even in incomplete second metacarpals or those affected by taphonomic damage (Gilmour et al. 2021). Because it only requires basic X‐ray equipment, radiogrammetry is especially suited for fieldwork and laboratories located near excavation sites and the sample's origin country, eliminating the need to transport remains to distant facilities or across borders. Its simplicity, affordability, and reproducibility make it an ideal method for cross‐population comparisons and large‐scale studies without reliance on high‐cost imaging technologies (Haara et al. 2006). As a result, metacarpal radiogrammetry continues to be widely used in clinical and paleopathological research. It enhances our understanding of bone loss in historical contexts and enables comparative analyses across diverse past populations (e.g., Umbelino et al. 2019; Wesp and Hernández 2022). Beyond its bioarchaeological and forensic applications, the metacarpal cortical area has also been explored as a potential tool for age estimation (e.g., Curate et al. 2022), based on its correlation with age‐related bone loss and other skeletal aging markers (e.g., Rissech et al. 2018). In a clinical context, such as the Framingham Osteoporosis Study, it was found that although metacarpal cortical area showed a modest association with hip fracture risk in men, it did not significantly predict risk in women, leading to the conclusion that it is not a strong or reliable predictor overall (Kiel et al. 2001). Despite these advancements, the relationship between cortical bone loss and stressor indicators remains underexplored. Most existing research has concentrated on BMD, with comparatively little attention given to cortical bone thinning specifically (e.g., Inagaki et al. 2001; Damanaki et al. 2024). Notably, Kaye et al. (2016) found that metacarpal cortical bone loss may be associated with tooth loss in men, suggesting that systemic bone loss could contribute to mandibular weakening and subsequent tooth loss, and Wactawski‐Wende (2001) associated the bone loss with periodontal disease. Although these findings are promising, the broader connection between bone loss and stressor indicators such as linear enamel hypoplasia (LEH) has never been addressed.

LEH has been widely used to study health outcomes during major biocultural transitions, such as shifts in food production and the adoption of agriculture (Cook and Buikstra 1979; Tomczyk et al. 2012) and the dietary changes that suppose weaning (Saunders and Keenleyside 1999). Various stressors can cause LEH, including malnutrition, deficiencies in calcium and vitamin D, systemic illnesses like hypothyroidism and rickets, infections, localized trauma, hereditary disorders, toxic exposures, and genetic or epigenetic factors (Nikiforuk and Fraser 1981; Hillson 2014; Kinaston et al. 2019). Dental enamel hypoplasia reflects developmental disturbances during early life. Specifically, it refers to a reduction in enamel thickness due to disruptions in ameloblast function during the secretory phase of enamel formation (Goodman et al. 1987; Lukacs 2001; Xing et al. 2015; Lewis 2018). These enamel defects are generally categorized into four types: pit‐form (PEH), plane‐form, linear‐form (LEH), and localized hypoplasia (Pindborg 1970; Towle and Irish 2020). The current study focuses on linear enamel hypoplasia (LEH), the most commonly observed form in archaeological samples (Guatelli‐Steinberg et al. 2004), given the difficulty of reliably identifying pitting or irregular enamel defects in macroscopic examination (Amoroso et al. 2014). LEH manifests as horizontal grooves or lines of diminished enamel on the crown surface of teeth (Towle and Irish 2020). While its precise etiology remains debated, LEH is widely recognized as a marker of “non‐specific stress” and has been linked to a variety of systemic disturbances, including malnutrition, hereditary disorders, vitamin deficiencies, environmental stressors, and even localized trauma (Huss‐Ashmore et al. 1982; Kanchan et al. 2015; Bereczki et al. 2018; Towle and Irish 2020).

Beyond functioning as an early life stress marker, research on LEH provides a valuable framework for investigating two key models of human biological responses to early‐life stress: (i) the predictive adaptive response and (ii) the plasticity/constraint models within life history theory (Morquecho et al. 2025). (i) The predictive adaptive response, also known as the thrifty phenotype hypothesis, suggests that mismatches between the prenatal environment and postnatal conditions can trigger physiological adjustments that improve survival during early life. However, if postnatal conditions differ from prenatal predictions, these adaptations may carry long‐term costs for health or development (Gluckman et al. 2005, 2007). These adaptations, if they exist, will correspond to early life programming (experiences and conditions early in life can shape how the body and brain develop, affecting health, behavior, and abilities later in life) (Langley‐Evans and McMullen 2010). In contrast, (ii) the plasticity/constraint perspective emphasizes that early‐life investments in maintenance and survival can delay or limit subsequent growth and reproductive output, reflecting trade‐offs that are central to life history strategies (Charnov 1991, 1993).

Importantly, because enamel does not remodel after formation, LEH serves as a permanent record of early‐life stressors in archaeological human remains. Moreover, nonspecific stress markers like porotic hyperostosis, LEH, and cribra orbitalia do not pinpoint a particular cause; rather, they show that the person experienced physiological stress throughout their life, and as a result, researching these markers provides important information about the well‐being and experiences of past populations (Casna and Schrader 2024).

Cortical bone loss typically, though not exclusively, occurs later in life and is closely associated with aging and other chronic or degenerative conditions (Hunter and Sambrook 2000; Sfeir et al. 2022). While no direct causal link between LEH and cortical bone loss has been firmly established in the literature, both may reflect systemic physiological and environmental stresses, such as malnutrition or metabolic imbalances, which affect enamel formation in childhood and bone integrity in adulthood.

Therefore, the aim of this study is twofold: (i) the first aim is, by using radiogrammetric analysis of the second metacarpal, to identify long‐term trends in cortical bone loss according to age, sex, and historical period, from the Roman era through the modern period in Barcelona, and to compare these trends with those documented in previous studies of past European populations. (ii) The second aim is to evaluate whether early‐life stressors influence bone health later in life by comparing LEH and MCI values among individuals of the same age group, sex, and historical period, and determining whether those with LEH exhibit lower MCI than their counterparts without the condition.

## 2 Material and Methods

### 2.1 Material

**FIGURE 1:**
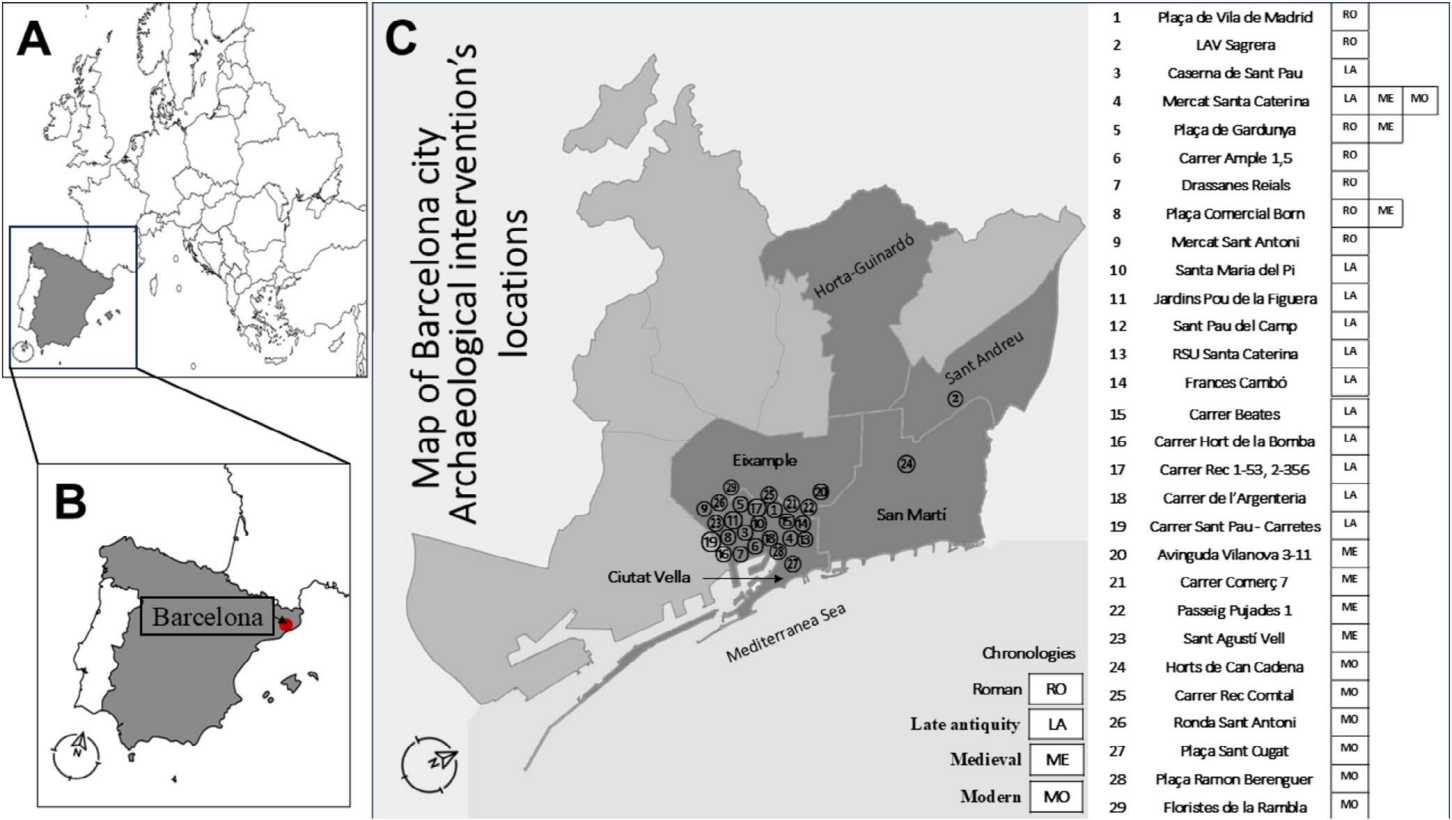
(A) Map of Europe showing the location of Spain (gray) and Portugal (white) in the Iberian Peninsula. (B) Enlarged view of the same map, highlighting the location of Barcelona in Spain. (C) Map of the city of Barcelona highlighting the location of the 29 different sites from which the material for this study was sourced.

The material analyzed in this study originates from the area now known as the city of Barcelona, located in the northeastern part of the Iberian Peninsula, in the central area of the Catalonian coast (Figure 1A,B). This material comes from 38 archaeological excavations in the city of Barcelona between 1984 and 2019. These excavations took place across 29 different sites located in four districts of Barcelona (Ciutat Vella, Eixample, Sant Martí, and Sant Andreu) (see Figure 1C). The excavations span four major historical periods of the city: Roman (1st–4th centuries CE), Late Antiquity (5th–7th centuries CE), Medieval (8th–14th centuries CE), and Modern (15th–18th centuries CE). The human sample from these 38 excavations consists of 694 individuals found in articulated positions (see Table S1). Skeletal remains are currently housed in the collections department of the Barcelona History Museum (MUHBA). Information on the socioeconomic status of the archaeological assemblages included in this study is not available in a consistent or comparable manner. Although some archaeological assemblages have published interpretations or inferences regarding socioeconomic context, these assessments are uneven and not based on uniform criteria. For this reason, socioeconomic status was not included as an analytical variable in this study. For detailed information about the archaeological excavations included in this study, please also refer to Table S1.

### 2.2 Methods

#### 2.2.1 Biological Profile and Second Metacarpal Selection Criteria

From these archaeological samples coming from Barcelona, only adult individuals with a well‐preserved second metacarpal bone were selected. Individuals exhibiting pathologies that could affect the analysis of the second metacarpal were excluded. Commingled or poorly preserved remains, as well as those for whom age and sex could not be reliably estimated, were also excluded from the study. A second metacarpal was eligible for the study if it was well‐preserved, with no broken or missing fragments, no observable taphonomic damage or erosion, and no pathological conditions such as fractures or synostosis with carpal bones that might compromise its morphology or internal bone integrity. Preference was given to the left metacarpal because the left side is preferred in osteological studies and clinical research, as many studies conclude the impact of handedness (dominant hand) on bone density and cortical thickness, supporting the idea that the preferential use of the dominant hand can influence bone characteristics (S. Mays 2002; Kekilli et al. 2006; Troy et al. 2021). Since this material is of archaeological origin, when the second left metacarpal was absent, the right one was used instead. Therefore, a total of 226 adults' second metacarpal bones were analyzed, comprising (*n* = 117 males; *n* = 109 females). Specifically, the sample included 48 individuals from the Roman period (*n* = 23 males; 25 females), 104 from Late Antiquity (*n* = 55 males; 49 females), 33 from the Medieval period (*n* = 20 males; 13 females), and 41 from the Modern period (*n* = 19 males; 22 females) (Table 1).

**TABLE 1:** Age and sex distribution of individuals with complete second metacarpals across different historical periods in this study.

Age category	Roman		Late antiquity		Medieval		Modern		Total	
	Males	Females	Males	Females	Males	Females	Males	Females	Males	Females
Young adult (21–30)	6	12	14	18	4	8	5	6	29	44
Middle adult (31–49)	14	11	32	24	15	5	12	14	73	54
Mature adult (50+)	3	2	9	7	1	0	2	2	15	11
Total	23	25	55	49	20	13	19	22	117	109
	48		104		33		41		226	

From these 226 adults, individuals with a mandible and a minimum of three in situ teeth were included in the LEH analysis: a central and a lateral incisor and a canine (please see subsection 2.2.3). Therefore, a subgroup of 153 individuals (*n* = 83 males; *n* = 70 females) was analyzed across four historical periods and the three age categories for the presence (LEH) or absence (non‐LEH) of LEH.

Individuals were classified into three age categories: Young adult (21–30 years), middle adult (31–49 years), and mature adult (50+ years). These categories were chosen for two main reasons. First, they reflect key biological stages of skeletal development and bone loss: the Young Adult group represents individuals who have reached peak bone mass but have not yet begun to experience significant age‐related bone loss; the Middle Adult group corresponds to the early onset of age‐related cortical bone thinning, and the Mature Adult group includes individuals at a higher risk for accelerated bone loss, decreased bone density, and increased skeletal fragility, commonly associated with aging and conditions such as osteoporosis. Second, this classification allows for direct comparison with previous studies that have used the same age groupings, thereby enhancing the consistency and comparability of findings (e.g., S. Mays 2006; Beauchesne and Agarwal 2014; Wesp and Hernández 2022).

Sex estimation was done by using the morphology of the innominate bones, cranium, and mandible (Ferembach et al. 1980; Brothwell 1981; Rissech and Malgosa 1991, 1997; Mitchell and Brickley 2017). Age estimation was estimated by using multiple indicators, including the pubic symphysis (Brooks and Suchey 1990; Todd 1920, 1921), auricular surface (Lovejoy et al. 1985), acetabulum (Rissech et al. 2006, 2019), ectocranial suture obliteration (Meindl and Lovejoy 1985), sternal ends of the fourth ribs (İşcan et al. 1984, 1985), sternal end of the clavicle (Falys and Prangle 2015), and patterns of dental wear (Brothwell 1981).

#### 2.2.2 Metacarpal Cortical Bone Loss Assessment

The second metacarpals were X‐rayed using an INTECH ForView radiography system, which consists of an INTECH FUTURA 32 X‐ray source and a digital detector INTECH CR SYSTEM2 with a pixel resolution of approximately 12‐line pairs per mm (a measure of spatial resolution) at Clínica Veterinaria Sagrada Familia. The digital radiographic images were analyzed using the MicroDICOM 2024.2 software version. The scale of each radiograph was calibrated using a 1 cm metal ruler, and the software ImageJ, version 1.48, was used to take the measurements. The second metacarpals were X‐rayed in an anteroposterior (AP) view and grouped into a set of between 40 and 45 metacarpals on a 43 × 35 cm^2^ plate. The posterior side of each second metacarpal was placed against the X‐ray plate, with the saddle of the proximal facet oriented perpendicularly to the plate following the corresponding orientations.

**FIGURE 2:**
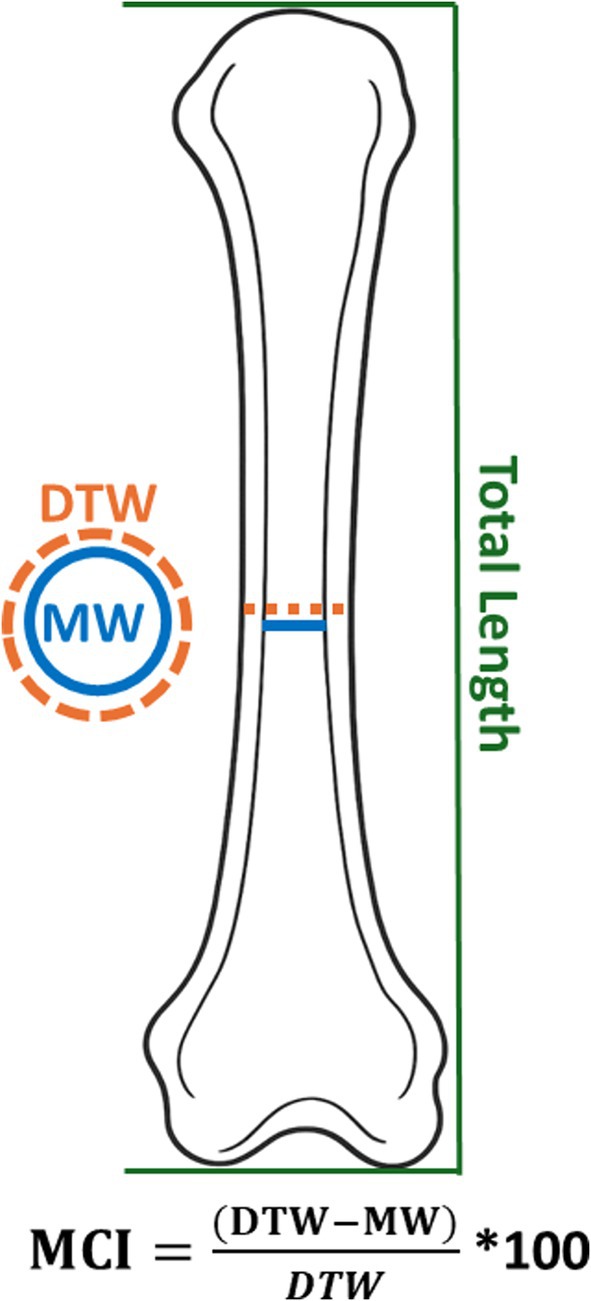
Illustration of the measurement locations used to calculate the metacarpal cortical index (MCI). The image shows the following measurements: Total length (green line), diaphyseal total width (DTW; orange line), and medullary width (MW; blue line), along with the formula used to calculate the MCI.

Cortical bone loss can be assessed by measuring the widening of the medullary (inner) cavity relative to the total width of the bone, which reflects the thinning of the cortical (outer) bone walls, an indicator quantified through metacarpal radiogrammetry (Ives and Brickley 2004). This technique involves measuring specific dimensions of the second metacarpal (Figure 2), including the diaphyseal total width (DTW) and the medullary width (MW), followed by calculating the metacarpal cortical index (MCI) (Figure 2) (Meema and Meema 1987; Ives and Brickley 2004). To ensure consistent measurement at the midshaft, the total length (TL) of the metacarpal is first recorded, and the midpoint (50% of TL) is identified. At this location, both DTW and MW were measured (Figure 2). The MCI is then calculated as the percentage ratio of cortical thickness defined as (DTW−MW) to the diaphyseal total width (DTW); see the formula for calculation of MCI in Figure 2.

#### 2.2.3 Linear Enamel Hypoplasia Criteria

This study employed a standardized protocol for evaluating the presence of LEH due to the difficulty of reliably identifying pitting or irregular enamel defects during macroscopic examination (Amoroso et al. 2014). LEH was identified based on the presence of well‐defined transverse grooves or bands of depressed enamel. A binary classification system was used: (1) absence (non‐LEH) and (2) presence (LEH) of at least one distinct linear enamel hypoplasia. All observations were conducted macroscopically by the first author to ensure consistency, using a 10× magnifying lens and oblique lighting to improve visibility and facilitate accurate identification of subtle defects.

Observations were limited to the mandibular permanent dentition, while maxillary dentition and isolated teeth were excluded to maintain consistency across samples. This choice is explained because in the assemblage most crania were highly fragmented or extremely fragile, often filled with sediment that could not be disturbed. Maxillae are particularly delicate, and teeth were frequently found isolated. In contrast, mandibles were generally better preserved and often retained teeth in situ, providing a reliable reference for assigning the precise anatomical position of teeth and allowing a standardized assessment.

**FIGURE 3:**
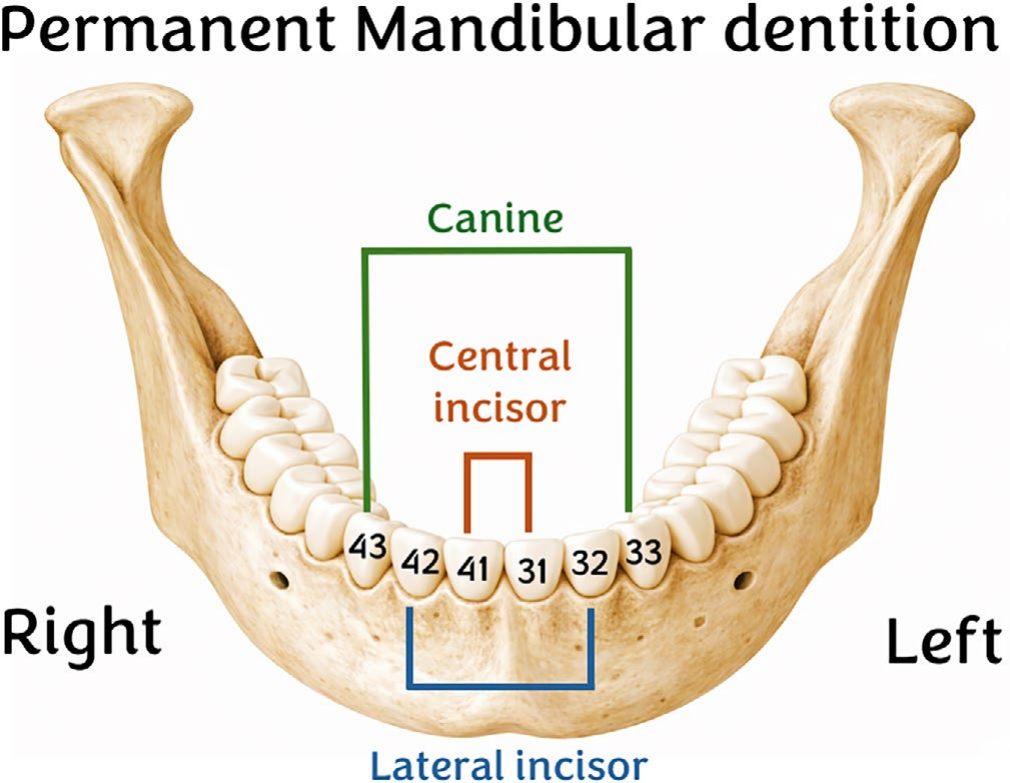
Odontogram of the anterior mandible and its dentition. The teeth included are the central incisors (41, right; 31, left), lateral incisors (42, right; 32, left), and canines (43, right; 33, left). The numbers correspond to the FDI World Dental Federation notation for each tooth.

Since incisors and canines are the teeth most likely to have enamel hypoplasia, LEH is typically evaluated on these teeth (Goodman and Armelagos 1985; Hillson 2014); for this study only mandibles with a minimum of three in situ teeth were included in the LEH analysis. To categorize LEH as present, at least one of each selected tooth type had to be observable: one central incisor, one lateral incisor, and one canine. According to the FDI World Dental Federation notation, these teeth correspond to 41 (right) or 31 (left) for the central incisor; 42 (right) or 32 (left) for the lateral incisor; and 43 (right) or 33 (left) for the canine (Figure 3).

These teeth are ideal for studying LEH because their enamel forms during early and middle childhood, a critical period for detecting early life stress, recording events from the twelfth week of prenatal development until 6 years of age after birth (Nanci 2013). Their long crowns and broad labial surfaces facilitate clear observation and measurement of hypoplastic lines. While bilateral symmetry can help distinguish systemic stress from localized damage, observations focused on either the right or left side due to frequent tooth loss or post‐mortem fragmentation in archaeological contexts.

Premolars and molars were excluded because their enamel forms later and over shorter periods, making them less reliable for detecting early life stress. In addition, their complex crown morphology and higher likelihood of post‐eruptive damage complicate accurate identification and interpretation of hypoplastic defects.

#### 2.2.4 Statistical Analysis

Regarding the metacarpal radiogrammetry analysis, the Kolmogorov–Smirnov test was applied to assess the normality of the distribution for each variable. When the data met the assumptions of normality, parametric tests were applied. When the data did not meet the assumptions of normality or the sample was very small, non‐parametric tests were applied.

As a first step, sex differences were assessed by Student's *t*‐test. Later, in order to evaluate differences across age, the total sample was analyzed under two conditions: (1) When both sexes were combined and (2) considering separated sexes, both taking into account the three age groups. In the first analysis, one‐way ANOVA was used to examine differences across age groups. If the ANOVA results were statistically significant, Tukey's post hoc test was conducted to identify specific group differences. In the second analysis, the Kruskal–Wallis test was applied, given the smaller sample sizes and potential deviations from normality. Further analyses were performed by stratifying the sample by sex, age group, and historical period. Within these stratified subgroups, due to the subsample size, the Kruskal–Wallis test was employed to detect differences in metacarpal measurements and cortical index.

To test the hypothesis regarding the association between the presence/absence of linear enamel hypoplasia and cortical bone loss, either Student's *t*‐test (for normally distributed variables) or the Mann–Whitney *U* test (for non‐normally distributed data) was used, depending on the distribution and characteristics of each comparison.

All statistical analyses were conducted using SPSS version 29.0 (SPSS Inc., Chicago, IL) and GraphPad Prism version 8.0 (GraphPad Software, San Diego, California, USA), with a significance level set at *p* < 0.05.

## 3 Results

### 3.1 Metacarpal Measurements and Cortical Index

As shown in Table 2, when analyzing the entire Barcelona sample, males, as expected, exhibit longer and wider metacarpals compared to females, with statistically significant sex differences observed in TL, DTW, and MW. However, despite females having a slightly higher mean MCI value than males, no significant sex differences were found (Table 2). A probable explanation for this lack of significance may be the inclusion of the entire sample, as confounding factors such as differences in age between individuals could have influenced the results.

**TABLE 2:** Mean bone measurements (in mm) and metacarpal cortical index for both sexes and *t*‐test values, considering the entire sample.

Sex	Total length (TL)		Diaphysis total width (DTW)		Medullary width (MW)		Metacarpal cortical index (MCI)	
	Mean	SD	Mean	SD	Mean	SD	Mean	SD
Males *n* = 117	69.92	0.41	8.69	0.07	4.13	0.10	52.65	10.07
Females *n* = 109	66.35	0.38	7.84	0.07	3.60	0.09	54.15	10.33
*T*‐test	*p* = 0.001*		*p* = 0.001*		*p* = 0.001*		*p* = 0.270	

* Indicates statistical significance at the *p* < 0.05 level.

Table 3 shows MCI by age, sex, and unisex group without considering time periods. Results indicated no significant differences for MCI across age groups in males, females, or when sexes were combined. Among the variables used to calculate MCI, only DTW showed significant age‐related differences (ANOVA *p* = 0.011; Young vs. Middle, Young vs. Mature, Tukey HSD) (see Table S2). DTW increased with age, with post hoc analysis indicating significant differences between the young and middle‐aged groups and between the young and mature groups. The other variables, total TL and MW, also tended to increase with age, reflecting medullary canal expansion, although these trends did not reach statistical significance (see Table S3). MCI decreased with age, consistent with the expansion of the medullary canal, but this change was also not statistically significant (Table 3). However, these non‐significant differences across all measurements and indices may be attributed to disparities in group sizes and inter‐period of time differences, which likely limit the statistical power of the analysis.

**TABLE 3:** Mean of metacarpal cortical index by age group and sex, including combined data, with Kruskal–Wallis and ANOVA test results.

Sex	Age groups	Metacarpal cortical index (MCI)	SD
Males	Young (*n* = 29)	53.23	7.60
	Middle (*n* = 73)	52.87	10.49
	Mature (*n* = 15)	50.41	12.35
	Kruskal–Wallis	*p* = 0.649	
Females	Young (*n* = 44)	55.28	10.88
	Middle (*n* = 54)	53.36	9.11
	Mature (*n* = 11)	53.48	13.95
	Kruskal–Wallis	*p* = 0.646	
Combined	Young (*n* = 73)	54.47	1.14
	Middle (*n* = 127)	53.08	0.88
	Mature (*n* = 26)	51.71	2.52
	ANOVA	*p* = 0.444	

Table 4 shows MCI between historical periods, taking into account sex and age groups. Results indicated that MCI did not exhibit any statistically significant differences across time periods in any of the age or sex categories (Table 4). Also, this lack of significant differences is observed in the variables utilized for the acquisition of the MCI (see Table S4). These findings suggest that despite some visible fluctuations in the MCI mean values, within each age and sex category, the historical period does not significantly influence the MCI (Table 4).

**TABLE 4:** Mean of the metacarpal cortical index divided by sex, age, and historical period groups.

Sex	Historical periods	Young adults				Middle adults				Mature adults			
		*n*	Mean	SD	Kruskal–Wallis	*n*	Mean	SD	Kruskal–Wallis	*n*	Mean	SD	Kruskal–Wallis
Males	Roman	6	54.14	7.71	*p* = 0.359	14	47.44	12.05	*p* = 0.301	3	38.91	10.35	*p* = 0.204
	Late antiquity	14	51.38	7.59		32	54.06	11.00		9	55.32	12.24	
	Medieval	4	51.10	2.85		15	55.47	8.31		1	51.58	—	
	Modern	5	59.04	8.75		12	53.47	8.73		2	45.00	7.07	
Females	Roman	12	53.68	11.04	*p* = 0.095	11	50.27	9.76	*p* = 0.527	2	53.53	12.60	*p* = 1.000
	Late antiquity	18	54.74	10.63		24	55.23	9.04		7	53.39	16.13	
	Medieval	8	63.24	8.48		5	54.94	5.06		—	—	—	
	Modern	6	49.48	10.73		14	52.03	9.75		2	53.76	15.03	

Table 5 shows the mean MCI values stratified by age group, sex, and time period and compares the results of the present study from Barcelona with previously published data from Italy, the United Kingdom, and Portugal. Overall, individuals from Barcelona across all four historical periods tend to exhibit similar or even higher mean MCI values for both males and females compared to these three European populations (Table 5). This trend is particularly pronounced in the Modern group, where MCI values for both sexes are notably higher than those reported in comparable cohorts from earlier studies. It is important to note, however, that many of the previous studies were based on relatively small sample sizes within each sex and age group, which may limit the robustness of direct comparisons (Table 5).

**TABLE 5:** Means of metacarpal cortical index (MCI) by sex, age, and historical period across various European countries.

Sex	Population/country	Chronology	Young adults		Middle adults		Mature adults		References
			*n*	Mean	*n*	Mean	*n*	Mean	
Males	Muge shell middens (Portugal)	Mesolithic (8400–5080 bce)	5	49.81	11	51.76	4	51.86	(Umbelino et al. 2019)
	Velia (Italy)	Roman (1st–2nd century CE)	6	53.13	20	51.24	13	41.51	(Beauchesne and Agarwal 2014)
	Barcelona (Spain)	Roman (1st–4th century CE)	6	54.14	14	47.44	3	38.91	Present Study
	Barcelona (Spain)	Late antiquity (5th–7th century CE)	14	51.38	32	54.06	9	55.32	Present Study
	Wharram Percy (United Kingdom)	Medieval (11th–16th century CE)	10	42.90	29	45.40	34	40.40	(S. Mays 1996)
	Barcelona (Spain)	Medieval (8th–14th century CE)	4	51.10	15	55.47	1	51.58	Present Study
	Spitalfields (United Kingdom)	Modern (19th century CE)	6	46.80	21	46.30	64	42.00	(S. Mays 2015)
	Barcelona (Spain)	Modern (15th–18th century CE)	5	59.04	12	53.47	2	45.00	Present Study
	Coimbra Identified Skeletal Collection (Portugal)	Modern (20th century CE)	14	54.11	33	56.79	67	51.77	(Umbelino et al. 2019)
Females	Muge shell middens (Portugal)	Mesolithic (8400–5080 BCE)	6	54.14	4	49.55	4	48.33	(Umbelino et al. 2019)
	Velia (Italy)	Roman (1st and 2nd century CE)	7	55.11	15	49.73	10	38.43	(Beauchesne and Agarwal 2014)
	Ancaster (United Kingdom)	Roman (3rd–4th century CE)	11	51.80	12	47.00	16	34.00	(S. Mays 2006)
	Barcelona (Spain)	Roman (1st–4th century CE)	12	53.68	11	50.27	2	53.53	Present Study
	Barcelona (Spain)	Late antiquity (5th–7th century CE)	18	54.74	24	55.23	7	53.39	Present Study
	Wharram Percy (United Kingdom)	Medieval (11th–16th century CE)	15	49.50	27	44.40	23	41.50	(S. Mays 1996)
	Barcelona (Spain)	Medieval (8th–14th century CE)	8	63.24	5	54.94	—	—	Present Study
	Barcelona (Spain)	Modern (15th–18th century CE)	6	49.48	14	52.03	2	53.76	Present Study
	Coimbra Identified Skeletal Collection (Portugal)	Modern (20th century CE)	15	55.19	30	55.47	60	42.96	(Umbelino et al. 2019)

In the majority of the compared samples (3 males and 6 female samples), peak bone mass was reached during young adulthood (Table 5). However, there are some samples (3 male and 3 female samples) that display the highest MCI during middle adulthood rather than in youth (Table 5), and in 2 male samples, the peak bone mass was reached in mature adulthood (Table 5).

### 3.2 Linear Enamel Hypoplasia

Among the 226 adult second metacarpal bones analyzed, 153 displayed observable dentition, including 83 males and 70 females (Table 6). The sample spanned historical periods as follows (Table 6): 36 individuals from the Roman period (18 males, 18 females), 60 from Late Antiquity (32 males, 28 females), 26 from the Medieval period (16 males, 10 females), and 31 from the Modern period (17 males, 14 females).

**TABLE 6:** Age and sex distribution of individuals with complete second metacarpals and observable dentition across different historical periods in this study.

Age category	Roman		Late antiquity		Medieval		Modern		Total	
	Males	Females	Males	Females	Males	Females	Males	Females	Males	Females
Young adult (21–30)	5	7	11	12	4	6	5	5	25	30
Middle adult (31–49)	10	9	12	11	12	4	10	8	44	32
Mature adult (50+)	3	2	9	5	—	—	2	1	14	8
Total	18	18	32	28	16	10	17	14	83	70
	36		60		26		31		153	

Table 7 shows the distribution and frequencies of presence and absence of linear enamel hypoplasia, considering age, sex, and historical periods. Results showed that the proportion of individuals with LEH varied across age and period but was consistently observed in both sexes (Table 7). Among males, LEH was most frequently observed in the young and middle adult categories, particularly during the Late Antiquity and Medieval periods (Table 7). In contrast, females showed a higher proportion of LEH in younger adults during the Roman and Late Antiquity periods (Table 7), with very few mature adult females affected.

**TABLE 7:** Distribution and frequencies of individuals with the presence of linear enamel hypoplasia (LEH) and absence (non‐LEH) by age groups, sex, and historical periods.

Sex	Period	Young adult (21–30)			Middle adult (31–49)			Mature adult (50+)			Combined		
		*n*	Non‐LEH	LEH	*n*	Non‐LEH	LEH	*n*	Non‐LEH	LEH	Total	Non‐LEH	LEH
Males	Roman	5	2 (40.0%)	3 (60.0%)	10	5 (50.0%)	5 (50.0%)	3	2 (66.7%)	1 (33.3%)	18	9 (50.0%)	9 (50.0%)
	Late antiquity	11	5 (45.5%)	6 (54.6%)	12	7 (58.3%)	5 (41.7%)	9	8 (88.9%)	1 (11.1%)	32	20 (62.5%)	12 (37.5%)
	Medieval	4	2 (50.0%)	2 (50.0%)	12	6 (50.0%)	6 (50.0%)	0	0 (0.0%)	0 (0.0%)	16	8 (50.0%)	8 (50.0%)
	Modern	5	5 (100.0%)	0 (0.0%)	10	7 (70.0%)	3 (30.0%)	2	2 (100%)	0 (0.0%)	17	14 (82.4%)	3 (17.7%)
	Total	25	14 (56.0%)	11 (44.0%)	44	25 (56.8%)	19 (43.2%)	14	12 (85.7%)	2 (14.3%)	83	51 (61.5%)	32 (38.6%)
Females	Roman	7	5 (71.4%)	2 (28.6%)	9	5 (56.6%)	4 (44.4%)	2	2 (100%)	0 (0.0%)	18	12 (66.7%)	6 (33.3%)
	Late antiquity	12	6 (50.0%)	6 (50.%)	11	8 (72.7%)	3 (27.3%)	5	3 (60.0%)	2 (40.0%)	28	17 (60.7%)	11 (39.3%)
	Medieval	6	2 (33.3%)	4 (66.7%)	4	2 (50.0%)	2 (50.0%)	0	0 (0.0%)	0 (0.0%)	10	4 (40.0%)	6 (60.0%)
	Modern	5	3 (60.0%)	2 (40.0%)	8	7 (87.5%)	1 (12.5%)	1	1 (100%)	0 (0.0%)	14	11 (78.6%)	3 (21.4%)
	Total	30	16 (53.3%)	14 (46.7%)	32	22 (68.8%)	10 (31.3%)	8	6 (65.0%)	2 (25.0%)	70	44 (62.9%)	26 (37.1%)
Combined	Roman	12	7 (58.3%)	5 (41.7%)	19	10 (52.6%)	9 (47.4%)	5	4 (80.0%)	1 (20.0%)	36	21 (58.3%)	15 (41.7%)
	Late antiquity	23	11 (47.8%)	12 (52.2%)	23	15 (65.2%)	8 (34.8%)	14	11 (78.6%)	3 (21.4%)	60	37 (61.7%)	23 (38.3%)
	Medieval	10	4 (40.%)	6 (60.0%)	16	8 (50.0%)	8 (50.0%)	0	0 (0.0%)	0 (0.0%)	26	12 (46.15%)	14 (53.9%)
	Modern	10	8 (80.0%)	2 (20.0%)	18	14 (77.8%)	4 (22.2%)	3	3 (100%)	0 (0.0%)	31	25 (80.7%)	6 (19.4%)
	Total	55	30 (54.6%)	25 (45.5%)	76	47 (61.8%)	29 (38.2%)	22	18 (81.8%)	4 (18.2%)	153	95 (62.1%)	58 (37.9%)

In the late antiquity period, both sexes showed similar percentages of individuals with LEH (37.5% males and 39.3% females) (Table 7), with representation across all age groups. Notably, mature adults were scarce in general, and particularly in the Medieval or Modern periods. In these two periods there are only two male individuals in the Medieval period and 1 female individual in the Modern period, and these three individuals did not show LEH. This lack of mature adults could indicate that most of them had already died before mature age, or perhaps the sample is simply small.

Across all periods, the proportion of individuals with LEH decreased with age, particularly among females. For example, in the female sample, LEH was present in 14 of 30 young adults (46.7%), 10 of 32 middle adults (31.3%), and only 2 of 8 mature adults (25.0%) (Table 7). A similar trend was observed among males, though slightly less pronounced. This pattern suggests that individuals with LEH may have experienced reduced survivorship into older age categories, potentially due to underlying health vulnerabilities associated perhaps with early‐life stressors.

**FIGURE 4:**
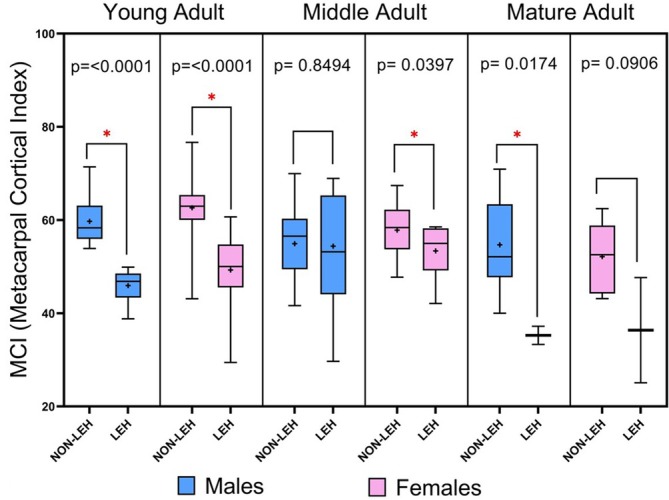
Box plot of the MCI (metacarpal cortical index) values by non‐LEH versus LEH status, considering sex (blue = males, pink = females) and age group. The mean values, SD, and *n* can be found in Table 8. Asterisks (*) indicate significant differences between groups (*p* < 0.05). Mean values are marked with a plus sign (+).

To increase the sample size, in Table 8 all the periods were considered together in order to observe the frequency of non‐LEH and LEH in each age group considering MCI of this age group. Overall, the combined data show that 58 out of 153 individuals (37.91%) exhibited LEH, with higher frequencies in the younger age groups (Table 8). Figure 4 clearly shows that individuals exhibiting (LEH) tend to have lower (MCI) values compared to those without this enamel defect. These differences are statistically significant in all ages and sexes with the exception of middle adult males and mature adult females (Figure 4).

**TABLE 8:** Mean cortical index values by sex and age group, comparing individuals with mild cognitive impairment (MCI) and the presence (LEH) or absence (non‐LEH) of linear enamel hypoplasia.

Sex	Age groups	*n*	Non‐LEH	Mean	SD	LEH	Mean	SD	Statistics	
Males (*n* = 83)	Young adult (21–30)	25	14	56.0	4.83	11	45.91	3.26	*t* = 8.118	*p* = 0.0001*
	Middle adult (31–49)	30	16	54.92	7.60	14	54.39	10.62	*t* = 4.674	*p* = 0.0001*
	Mature adult (50+)	44	25	57.82	5.44	19	53.36	5.47	*t* = 0.1910	*p* = 0.8494
Females (*n* = 70)	Young adult (21–30)	32	22	62.60	7.38	10	49.27	8.24	*t* = 2.150	*p* = 0.0397*
	Middle adult (31–49)	14	12	54.68	9.60	2	35.25	2.76	*U* = 2.757	*p* = 0.0174*
	Mature adult (50+)	8	6	52.16	7.74	2	36.35	15.95	*U* = 2.014	*p* = 0.0906

*Note:* Statistical tests were performed using Student's *t*‐test (*t*) and the Mann–Whitney *U* test (*U*).

* Indicate significant differences between groups (*p* < 0.05).

Regarding the analysis of differences in MCI between individuals with and without LEH, notable patterns emerged across sex, age categories, and historical periods (Table 9), showing that individuals with LEH have lower MCI values than those with non‐LEH. However, these differences were not significant, except in the young adult group (21–30 years) of Late Antiquity males, which showed significant differences (Table 9), and in females of this period, which were near significance (Table 9). This lack of differences perhaps is due to the division of the sample into subsamples, which diminishes the sample size.

**TABLE 9:** Means of MCI (metacarpal cortical index) along with standard deviation, Mann–Whitney *U* test (*U*) and *p* values, grouped by LEH status (non‐LEH vs. LEH), age group, sex, and historical period.

Age categories	Sex	Period	Non‐LEH			LEH			*U*	*p*
			*n*	Mean	SD	*n*	Mean	SD		
Young Adult (21–30)	Male	Roman	2	59.83	5.01	3	46.69	3.34	6.00	0.20
		Late antiquity	5	60.80	7.07	6	45.45	3.82	30.00	0.00*
		Medieval	2	59.76	4.59	2	46.14	2.73	4.00	0.33
		Modern	5	59.19	3.69	—	—	—	—	—
	Female	Roman	5	64.90	5.15	2	48.20	2.63	10.00	0.10
		Late antiquity	6	62.65	10.90	6	54.35	4.90	30.00	0.07*
		Medieval	2	58.34	6.56	4	39.81	7.37	8.00	0.13
		Modern	3	61.54	2.02	2	54.05	5.73	6.00	0.20
Middle Adult (31–49)	Male	Roman	5	49.48	6.40	5	57.71	10.68	7.00	0.31
		Late antiquity	7	50.46	7.43	5	54.87	9.06	12.00	0.43
		Medieval	6	49.48	6.40	6	57.71	10.68	7.00	0.31
		Modern	7	60.43	6.25	3	63.33	4.99	8.50	0.73
	Female	Roman	5	58.13	5.23	4	51.54	7.25	15.50	0.22
		Late antiquity	8	57.31	4.15	3	56.31	2.00	15.00	0.63
		Medieval	2	57.62	13.83	2	55.77	3.37	2.00	1.00
		Modern	7	58.24	5.71	1	46.92	—	7.00	0.25
Mature Adult (50+)	Male	Roman	2	50.00	0.00	1	37.20	—	2.00	0.48
		Late antiquity	8	58.07	9.66	1	33.30	—	8.00	0.22
		Medieval	—	—	—	—	—	—	—	—
		Modern	2	45.79	8.19	—	—	—	—	—
	Female	Roman	2	53.53	12.60	—	—	—	—	—
		Late antiquity	3	54.25	4.63	2	36.35	15.95	6.00	0.20
		Medieval	—	—	—	—	—	—	—	—
		Modern	1	43.14	—	—	—	—	—	—

* Indicate significant differences between groups (*p* < 0.05).

Notably, no young adult males with LEH were recorded in the modern period, precluding comparisons in that subgroup. Among mature adults (50+ years), sample sizes were extremely small, particularly in the Medieval and Modern periods. Nonetheless, some notable contrasts were observed. For instance, in late antiquity males, those with non‐LEH had a mean MCI of 58.07, while the sole male with LEH had a much lower MCI of 33.30.

## 4 Discussion

This study has examined long‐term trends in cortical bone loss among the historical population of Barcelona, covering a period from the 1st to the 18th century CE, by using radiogrammetry of the second metacarpal. Additionally, in order to explore whether LEH is associated with reduced cortical bone mass in adulthood, the study has compared MCI values between individuals with and without LEH, a non‐specific marker of early life stress. To our knowledge, this is the first paleopathological study in Spain to investigate cortical bone loss through radiogrammetry in an archaeological context and the first in the broader literature to explore the relationship between MCI and LEH.

As a first approximation, the sexual differences of each variable were examined. As expected, significant sexual differences were observed in TL, DTW, and MW (Table 2) with higher values in males, indicating that males tend to exhibit longer and wider bones than females. These differences are in accordance with prior research on metacarpal morphology (e.g., Scheuer and Elkington 1993; Barrio et al. 2006; Curate et al. 2019; Wesp and Hernández 2022). In general, males have larger bones and experience longer periods of skeletal maturation, supported by higher testosterone levels, which enhance periosteal apposition and result in greater cortical robustness (Seeman 2002; Doyle et al. 2011). Conversely, female skeletal growth tends to favor endosteal over periosteal bone deposition, likely an adaptation to reproductive physiology that limits external bone expansion (Bass et al. 1999; Seeman 2008; Gosman et al. 2011).

### 4.1 Sex, Age, and Period‐Related Differences in Bone Loss

Significant differences in MCI between age groups, either in the combined sample (unisex) divided by age (Table 2) or between sexes within specific age groups and periods (Tables 3 and 4), were not observed. Perhaps, these observations may be explained by progressive bone apposition in specific regions of the skeleton (MacLaughlin and Bruce 1986), in this case the second metacarpal, which will be accompanied by endosteal resorption of this bone. The lack of statistical significance may be partially explained by small sample sizes in certain groups, especially among mature adults (e.g., *n* = 1–3 in some groups), which reduces the power to detect differences. Overall, the data do not support a strong temporal trend in MCI across the Roman to Modern periods when accounting for age and sex.

Instead, MCI seems more closely related to age‐related bone loss, as suggested by the observed trend to decrease with age in Table 3, even though these trends did not reach statistical significance. Any potential changes in cortical thickness over time are likely minor or confounded by other factors such as lifestyle, pre‐existing pathological conditions, or broader environmental influences.

Overall, individuals from Barcelona across all four historical periods tend to exhibit similar or even higher mean MCI values for both males and females compared to other European populations. This trend is particularly pronounced in the modern group, where MCI values for both sexes are notably higher than those reported in comparable cohorts from earlier studies. It is important to note, however, that many of the previous studies were based on relatively small sample sizes within each sex and age group, which may limit the robustness of direct comparisons (Table 5). In general, in these samples, the highest MCI value is obtained in middle adults. This pattern is also observed in the medieval males of Wharram Percy (Table 5) from the United Kingdom, in which the sample size of mature adults is relatively high. This prompts the consideration that, probably, in past populations, like in current populations, there would have been differences in susceptibility to bone loss and osteoporosis between populations, since differences have been observed between contemporary populations (Lunt et al. 1997; Johnell and Kanis 2004). Several factors may explain these population differences, such as physical activity, nutrition, and environment. Physical activity is known to stimulate periosteal bone formation, which can help mitigate age‐related bone loss (Schmitt et al. 2007; S. A. Mays 2016). Similarly, adequate nutrition and sun exposure, especially sufficient calcium intake during childhood and enough vitamin D, are crucial for achieving optimal peak bone mass and maintaining skeletal health throughout life (Schaafsma et al. 1987; Ruff et al. 2015). Portugal and Spain are in Southern Europe, and due to their latitude, they receive high levels of solar radiation throughout the year, which enables residents to produce vitamin D more effectively through skin exposure to sunlight. In addition, studies based on faunal remains and stable isotopes indicate that the individuals from Barcelona in the past had, in general, an adequate nutritional intake, having access to a C_3_ terrestrial diet based on domestic animals (Rissech et al. 2016; Salazar‐García et al. 2022). Mostly, health markers such as life expectancy at birth, mean stature, and mean weight indicated good health in the different historical populations of Barcelona analyzed in the present study (e.g., Cevallos et al. 2023; Merino and Rissech 2025; Cevallos and Rissech, manuscript under review). However, among the periods analyzed in Barcelona, three stand out: two due to sociopolitical conflicts (late antiquity and early medieval period) and the third due to the introduction of new crops (modern period) (Merino and Rissech 2025; Cevallos and Rissech, manuscript under review).

Late antiquity (4th–8th centuries CE) coincides with the collapse of Roman political structures and the subsequent transition to Visigothic and, later, Islamic rule. These centuries were characterized by sociopolitical fragmentation, administrative realignment, and significant economic transformation, all of which likely contributed to increased social uncertainty and uneven access to essential resources (Beltrán de Heredia 2010). Despite some continuity in dietary staples, particularly the continued reliance on C_3_ cereals such as wheat and barley, archaeobotanical and isotopic evidence points to a decline in the consumption of animal protein during this period (Fuller et al. 2010; Jordana et al. 2019; García‐Moreno et al. 2024). This likely reflects broader socioeconomic constraints, including changes in food production, trade networks, and social organization, which may have limited dietary diversity, especially for lower‐status populations. Supporting this interpretation, anthropometric indicators reveal subtle but telling biological responses to environmental stress. Average stature and body mass show slight declines compared to the preceding Roman period, while variability in nutritional status increases, suggesting growing inequality in health outcomes and living conditions (Cevallos and Rissech, manuscript under review).

The early medieval period in Barcelona (9th–10th centuries CE) experienced political instability because of the creation of the Spanish Mark (La Marca Hispanica) to form a buffer zone of Christian counties along the frontier with Islamic territory. Barcelona was captured several times by Islamic or Frankish attacks, of which there were a considerable number. During this period, the population of Barcelona experienced some food scarcity, such as, for example, fresh fruits, and vegetables (Merino and Rissech 2025). According to the results of the present study, what is most striking during the medieval period is the notable cortical bone robustness observed in young adult females, who exhibited the highest MCI values when compared both to other historical periods in Barcelona and to populations from other parts of Europe (Table 5). Possible explanations include differences in labor demands, timing of reproductive events, or selective nutritional advantages favoring females. It is also important to note that the medieval period in this study spans a broad timeframe, from the 9th to the 14th centuries, encompassing various historical phases, yet the sample size required grouping all these centuries together. Overall, this sex‐based difference in physiological development highlights the complex ways biological systems respond to historical stressors.

In the Modern period (15th–18th centuries CE), one of the most significant developments was the diversification of the diet. The introduction and eventual implementation of New World crops, especially tobacco, cocoa beans, potatoes, tomatoes, haricot, peanuts, maize, beans, pumpkin, pineapple, and the turkey, among others, helped buffer food insecurity and provided more consistent caloric intake among the lower classes (McNeill 2010; Buchanan et al. 2024). Alongside shifts in agricultural production and market integration, these dietary changes likely contributed to a broad‐based enhancement in nutritional status. Bioanthropological data from this period support this interpretation: markers of childhood physiological stress declined, life expectancy began to rise, and average body weight increased, especially among children and women, without a corresponding rise in obesity (Cevallos and Rissech, manuscript under review). These improvements were particularly evident in the male population. Modern males displayed the highest MCI values of all demographic groups studied, indicating enhanced cortical bone development and suggesting that improved early‐life nutrition and reduced chronic stress played a pivotal role in shaping skeletal outcomes (Santonja 1999; Floristán 2011). In contrast, females did not reach peak MCI in young adulthood, which may reflect persistent structural inequalities in access to resources or the lingering impact of sex‐specific labor demands and reproductive stressors. The divergence between male and female skeletal trajectories in this period underscores how unevenly the benefits of modernization were distributed and highlights the importance of examining sex as a mediating factor in historical patterns of health and development.

Physical activity and nutrition influence bone metabolism differently depending on the anatomical site and the specific cortical envelope involved (Robling and Stout 2004; Peck and Stout 2007; Gosman et al. 2011). There is also evidence that mechanical stress and nutritional status affect male and female bone morphology differently (Gilsanz et al. 1997; Gosman et al. 2011). By encouraging bone growth during puberty and preserving bone health in adulthood, sex hormones have a major impact on skeletal development (Riggs et al. 2002). Males attain higher peak bone mass, larger bone size, and greater skeletal strength than females during puberty, demonstrating sexual differences in bone structure (Garn 1970; Seeman 2001; Callewaert et al. 2010). Interestingly, females in the Barcelona sample exhibited higher mean MCI values than males, a trend consistent across age groups and historical periods (Table 5). Similar findings from other past European populations suggest greater endosteal deposition in females (Table 5), possibly as an adaptation to reproductive demands (Martin 2003). This may explain the narrower medullary cavities observed in young females, although differences were not statistically significant (Table 5).

Across the sample, MCI peaked during young adulthood, consistent with clinical data showing that peak bone mass is typically achieved by the third decade of life (Böttcher et al. 2006). The subsequent decline in MCI is linked to age‐related hormonal changes, particularly decreased estrogen levels in postmenopausal women and reduced testosterone in aging men (Brown 2017; Almeida et al. 2017). Testosterone supports osteoblast activity and suppresses osteoclast function (Orwell et al. 2013; Wiren 2013), while estrogen deficiency accelerates bone resorption and contributes to cortical loss (Rauner et al. 2007; Cawthon et al. 2016), increasing osteopenia and osteoporosis risk (Szulc 2010).

Diachronic and geographical comparisons (Table 5) suggest that the patterns of age‐ and sex‐related cortical bone loss observed in the Barcelona sample are generally consistent with those found in other European skeletal collections (S. Mays 2000, 2006; Glencross and Agarwal 2011; Umbelino et al. 2019). Although statistically significant differences are not always detected in such studies, a general trend of declining MCI with increasing age is typically observed. However, this decline is often subtle and not always statistically significant when data are disaggregated by both sex and age, as noted in previous research (e.g., S. Mays 2015; Umbelino et al. 2019). Notably, the similarity of the Barcelona sample to Portuguese patterns may reflect shared environmental and lifestyle factors characteristic of southern European populations. These include more temperate climates, greater sunlight exposure, and historically better access to fresh produce, which likely contributed to less harsh living conditions compared to northern Europe.

Lastly, it is essential to recognize that bone aging is subject to significant populational and individual variability. Even within the same population, responses to biological and environmental stressors differ. Therefore, while broad patterns can be identified, they must be interpreted with sensitivity to historical, cultural, and individual diversity. This complex interplay of sex, age, and context should encourage researchers to analyze both structural and density‐based measures of bone health. A multidimensional approach is necessary to fully understand bone aging in historical populations.

### 4.2 Relationship Between the Presence of LEH and Cortical Bone Loss

The present study explored whether LEH is related to reduced metacarpal cortical index (MCI), a proxy for cortical bone thinning. Cortical bone thinning is a hallmark of bone loss and is influenced by hormonal changes, chronic diseases, malnutrition, and developmental factors (Seeman 2003; Ebeling et al. 2022). While teeth and bone differ in development, since teeth do not remodel after formation unlike bone, bioarchaeological evidence shows that LEH often co‐occurs with other skeletal pathologies, suggesting that systemic early‐life stress may affect both tissues (King et al. 2005; Temple 2007). Early life insults such as nutritional deficiencies, infectious diseases, or emotional trauma disrupt enamel formation and leave permanent LEH markers (Schwartz et al. 2006). Because enamel is durable and does not remodel, LEH provides reliable data on childhood growth disruptions with minimal bias, though its multifactorial etiology complicates interpretation (Guatelli‐Steinberg 2001; Schwartz et al. 2006; Ritzman et al. 2008).

The results of the present study did not find significant differences between the individual values of MCI in relation to the presence of LEH and non‐LEH when considering historical periods, sex, and age groups. This was probably due to the subsample sizes. However, when analyzing the entire sample without accounting for historical periods, statistically significant differences were observed (Figure 4, Table 8). Individuals exhibiting LEH tended to have lower MCI values compared to those without LEH, regardless of sex and age. Although the data does not yet confirm a completely direct relationship between the presence of LEH and cortical bone thinning, the findings suggest that early life stressors reflected by LEH may negatively impact bone health status in adulthood. These results are in accordance with current clinical epidemiological data that links early life stressors and disease in later life, suggesting that atopic disorders (Carrington and Langley‐Evans 2006), psychological traits (Indredavik et al. 2005), and osteoporosis (Cooper et al. 1997) may be subject to early life programming. As we explained in the Introduction section, the term “programming” refers to the idea that experiences and conditions early in life can shape how the body and brain develop, affecting health, behavior, and abilities later in life (Langley‐Evans and McMullen 2010). This can be explained by the rapid growth in the early stages of human development, in which the individual is very sensitive to environmental perturbations. The stressors can disrupt cellular differentiation and proliferation, leading to alterations in normal developmental pathways and impairing the maturation of organs and tissues (Langley‐Evans and McMullen 2010). These signals of disturbance may include (i) nutritional factors, (ii) psychological and physiological stressors, and (iii) imbalances in the endocrine pathway between mother and fetus (Seckl 2004). These stress factors seem to be associated with miscarriage and fetal death (Maconochie et al. 2007). However, when these disturbances occur, in order to ensure critical tissue functioning and survival, the developing individual must execute adaptive responses. Due to this adaptive response, and because the development involves the formation of well‐ordered key structures, this process of adaptation will likely result in irreversible modifications to tissue structure and function (e.g., altered cell types or altered number of cells and/or altered functional units) (Langley‐Evans and McMullen 2010). In the adult, these modifications can alter the capacity to modulate physiological function and susceptibility to disease (Langley‐Evans and McMullen 2010).

On the other hand, and from a broader perspective, LEH is also viewed as an indicator not only of hardship but also of individual resilience and survival (Hoover and Hudson 2016). The “osteological paradox” highlights that skeletons without pathology may belong to individuals who died quickly without recovery, whereas those showing lesions like LEH may represent survivors of repeated adversities (Wood et al. 1992). Since LEH develops gradually, its higher prevalence in adults can reflect the resilience of a population (Ortner 1991; Wood et al. 1992). However, to affirm any statement, more research is necessary.

### 4.3 Strengths and Limitations of the Study and Directions for Future Research

This study presents several notable strengths. Foremost is its substantial sample size of 226 individuals from archaeological contexts in Barcelona, which enhances the reliability and representativeness of the MCI results compared to previous studies with smaller cohorts. The inclusion of individuals from four historical periods—Roman, Late Antiquity, Medieval, and Modern—allows a diachronic analysis of cortical bone maintenance and loss spanning nearly two millennia (1st–18th centuries CE). This long temporal coverage provides insight into skeletal changes across different age and sex groups and reveals demographic patterns in bone loss over time. Importantly, this is the first study to examine cortical bone loss diachronically in a Spanish population, addressing a gap in European paleopathological literature and providing a benchmark for future comparative research. The study also introduces a novel investigation into the potential association between LEH, a marker of early‐life stress, and cortical bone loss using metacarpal cortical index (MCI) as a proxy for cortical thinning.

Several limitations should be considered. First, while the overall sample is large, certain subgroups, particularly mature adults segmented by sex and period, are relatively small. This limits statistical power and may affect the generalizability of age‐related findings, as metabolic conditions affecting bone are more likely to manifest in older individuals. Paleodemographic estimates for historical populations in Barcelona suggest that life expectancy at birth rarely exceeded 27 years (Cevallos and Rissech, manuscript under review), which likely accounts for the limited number of older adults in the sample, probably because these periods correspond to the pre‐antibiotic era, when fewer people lived to old age. In addition, the cross‐sectional nature of skeletal data restricts interpretations to a single moment in time, typically at death, and does not allow direct observation of physiological changes across the life course (Agarwal 2021). Although comparisons across historical periods provide valuable diachronic insight, they cannot substitute for longitudinal data.

Methodological limitations are also important. Radiogrammetry was employed to assess cortical bone thickness, a widely used and effective tool in both clinical and paleoanthropological research. However, it lacks the sensitivity to capture fine‐scale microarchitectural features, particularly within trabecular bone, which are essential for understanding metabolic bone diseases such as osteoporosis (Yasaku et al. 2009; Curate 2014). Radiogrammetry cannot assess intracortical resorption, porosity, or irregular endosteal scalloping, which are key indicators of high bone turnover (Jergas 2008), and it does not measure bone loss in regions most affected by fragility fractures (Agarwal 2018). Skeletal asymmetry related to handedness or habitual activity may also introduce bias, as the best‐preserved second metacarpal was selected when preservation differed between sides (Lazenby 2002; Glencross and Agarwal 2011).

Dental factors posed additional limitations. While the middle and cervical thirds of teeth were generally well‐preserved in young and middle‐aged adults, occlusal wear made recording the occlusal third challenging, reducing observations of LEH. Older individuals were underrepresented, with many exhibiting partial or complete edentulism, further limiting the sample available for examining late‐life bone and dental patterns (Kacki et al. 2025).

Finally, the broader interpretive challenges of paleopathology must be acknowledged. Archaeological skeletal samples represent only a fraction of past populations and may introduce bias in demographic reconstructions (Konigsberg and Frankenberg 2002; DeWitte and Stojanowski 2015). Hidden factors influencing observable bioarchaeological patterns, referred to as the osteological paradox, create inherent uncertainties that require careful theoretical consideration to avoid overgeneralization (De Luca et al. 2023; Anderson and DeWitte 2026).

Future studies could incorporate advanced imaging techniques capable of assessing trabecular architecture, cortical porosity, and other microstructural characteristics to provide deeper insight into metabolic bone disease in historical populations. Standardizing side selection and integrating biomechanical reconstructions where possible would improve accuracy and interpretability. Expanding the age range and exploring alternative markers of physiological stress may further enhance understanding of skeletal health across the life course.

## 5 Conclusions

This study presents the first long‐term assessment of cortical bone loss in a Spanish population, analyzing skeletal remains from Barcelona that span nearly 2000 years, from the 1st to 18th century CE. By evaluating the MCI across age groups, sexes, and historical periods, it reveals patterns of bone maintenance influenced by both biological and sociocultural factors.

The MCI trends observed in the Spanish sample are similar to those found in earlier Portuguese studies, which show peak bone mass in middle adulthood and only slight declines in later life. This suggests that differences in age‐related bone loss existed among past populations, much like they do today. These differences may be explained by factors such as physical activity, nutrition, vitamin D levels, and environmental conditions. Southern Europe's latitude provides abundant sunlight, supporting effective vitamin D synthesis, while archaeological and isotopic evidence indicates that people in ancient Barcelona had generally adequate diets based on domestic animals and a variety of plant foods.

An important finding of this study is the significant association between LEH and lower MCI values. This suggests that early‐life stressors may have long‐term consequences for adult bone health and supports the early life programming theory. Although this connection requires further validation, especially given the complex causes of LEH, it opens new avenues for research into the lifelong impact of early‐life stress.

To our knowledge, this is the first paleopathological study in Spain to investigate cortical bone loss through radiogrammetry in an archaeological context and the first to examine the relationship between MCI and LEH in any population. Despite certain limitations, including small sample sizes in some historical periods, this work lays the groundwork for future paleopathological research in the Iberian Peninsula and encourages comparative studies with other European populations.

Future research should include high‐resolution imaging, biomechanical modeling, and stable isotope analysis to expand our understanding of historical health patterns. Examining additional stress markers such as cribra orbitalia, porotic hyperostosis, and Harris lines, as well as exploring genetic relationships among individuals, could provide further insight into the lived experiences of people in the past. In conclusion, this study highlights the complex interaction between biology, sex, and historical change in shaping skeletal health over time. It demonstrates the value of a long‐term and multidisciplinary approach in reconstructing the health and lives of past populations.

## Author Contributions

**Antony Cevallos:** conceptualization, writing – original draft, writing – review and editing, data curation, formal analysis, methodology, investigation, software, validation, project administration, resources, funding acquisition, visualization. **Alexandre Tarragó:** methodology, resources, software, formal analysis. **Carme Rissech:** writing – original draft, writing – review and editing, supervision, methodology, investigation, funding acquisition, conceptualization, project administration, visualization, validation.

## Funding

This work was supported by Generalitat de Catalunya, SGR Evolució Social, Cultural i Biològica al Pleistocè (StEP), 2021 SGR 01239; Ministerio de Ciencia, Innovación y Universidades, PID2024‐156477NB‐C31; Agència de Gestió d'Ajuts Universitaris i de Recerca, 2023 FISDU 00247 and Universitat Rovira i Virgili.

## Conflicts of Interest

The authors declare no conflicts of interest.

## Supporting information


**Table S1:** Archaeological sites included in the investigation, listing the names, city's district, and codes of the interventions according to the Carta Arqueològica de Barcelona (Archaeological Chart of Barcelona). The table also includes the number of individuals analyzed in this study for each intervention, followed by their chronology, respective period, and the reference for the dating.
**Table S2:** Mean bone measurements (in mm) for the entire sample according to age categories, including ANOVA results and corresponding post hoc Tukey test values.
**Table S3:** Mean bone measurements (in mm) by age groups, divided by sex, with Kruskal–Wallis test values.
**Table S4:** Mean bone measurements (in mm) and metacarpal cortical index (in cm), divided by sex, age, and period groups, along with Kruskal–Wallis statistical test results.

## Data Availability

The data supporting the findings of this study are available from the first and corresponding authors upon reasonable request. The data are not publicly available due to privacy or ethical restrictions.
