# Supplementary material for: Cortical Bone Loss in Barcelona Across Time (1st Century CE–18th Century CE) and Its Potential Relationship With Linear Enamel Hypoplasia
Source: Am J Biol Anthropol. 2026 Apr 3;189(4):e70241. doi: 10.1002/ajpa.70241 (PMC13048880; doi:10.1002/ajpa.70241)
Supplement: Supplementary file 1 — Table S1: Archaeological sites included in the investigation, listing the names, city's district, and codes of the interventions according to the Carta Arqueològica de Barcelona (Archaeological Chart of Barcelona). The table also includes the number of individuals analyzed in this study for each intervention, followed by their chronology, respective period, and the reference for the dating. Table S2: Mean bone measurements (in mm) for the entire sample according to age categories, including ANOVA results and corresponding post hoc Tukey test values. Table S3: Mean bone measurements (in mm) by age groups, divided by sex, with Kruskal–Wallis test values. Table S4: Mean bone measurements (in mm) and metacarpal cortical index (in cm), divided by sex, age, and period groups, along with Kruskal–Wallis statistical test results. [file AJPA-189-e70241-s001.docx]

**Supplemental Material**

**Supplementary data table 1.**

Archaeological sites included in the investigation, listing the names, city’s district and codes of the interventions according to the Carta Arqueològica de Barcelona (Archaeological Chart of Barcelona). The table also includes the number of individuals analyzed in this study for each intervention, followed by their chronology, respective period, and the reference for the dating.

| No. | Archaeological intervention name | City  District | Archaeological intervention Code | Chronology by century (C.E.) | Period | References of the archaeological intervention report | Total of Adult Individuals | Total of Qualified Individuals for this study |
| --- | --- | --- | --- | --- | --- | --- | --- | --- |
| 1 | Plaça de Vila de Madrid | Ciutat Vella | 009/01 | 1st to 3rd | Roman | (Busquets et al., 2006) | 21 | 16 |
| 2 | LAV Sagrera Josep Soldevila | San Andreu | 106/08 | 3rd to 4th | Roman | (Balaguer, 2016) | 23 | 6 |
| 3 | Caserna de Sant Pau | Ciutat Vella | 302/90 | 4th to 6th | Late antiquity | (Granados and Blasco, 1990) | 21 | 10 |
| 4 | Mercat Santa Caterina | Ciutat Vella | 068/99; 001/01 | 4th to 6th | Late antiquity | (Huertas and Aguelo, 2006) | 66 | 20 |
|  |  |  |  | 11th to 14th | Medieval |  | 17 | 7 |
|  |  |  |  | 15th to 18th | Modern |  | 12 | 1 |
| 5 | Plaça de Gardunya | Ciutat Vella | 089/10 | 1st to 4th | Roman | (Velasco et al., 2017) | 1 | 1 |
| 6 | Carrer Ample 1, 5 | Ciutat Vella | 131/04; | 1st to 4th | Roman | (Triay, 2006) | 1 | 1 |
|  |  |  | 106/07 | 1st to 4th | Roman | (Prida, 2009) | 14 | 9 |
| 7 | Drassanes Reials de Barcelona | Ciutat Vella | 057/11 | 1st to 2nd | Roman | (Moreno and Esqué, 2013) | 17 | 4 |
|  |  |  | 013/10 | 1st to 3rd | Roman | (Ravotto, 2011) | 2 | 1 |
| 8 | Plaça Comercial Born | Ciutat Vella | 303/91 | 3rd to 4th | Roman | (Farré and Fernández, 1994) | 37 | 8 |
|  |  |  |  | 8th to 9th | Medieval |  | 14 | 7 |
| 9 | Mercat Sant Antoni | Eixample | 056/11, 003/12 | 1st to 2nd | Roman | (Hinojo, 2020) | 5 | 1 |
| 10 | Santa María del Pi | Ciutat Vella | 061/07 | 3rd to 4th | Late antiquity | (Miquel, 2010) | 1 | 1 |
| 11 | Jardins Pou de la Figuera | Ciutat Vella | 088/06 | 5th to 6th | Late antiquity | (Vilardell, 2009) | 27 | 5 |
| 12 | Sant Pau del Camp | Ciutat Vella | 306/89 | 4th to 5th | Late antiquity | (Bacaria et al., 1991) | 24 | 5 |
| 13 | RSU Santa Caterina | Ciutat Vella | 010/04 | 3rd to 8th | Late antiquity | (Bordas and Subiranas, 2006) | 15 | 7 |
|  |  |  | 137/02 | 3rd to 5th |  | (Camarasa and Piera, 2005) | 16 | 7 |

**Supplementary data table 1 (continued)**

| No. | Archaeological intervention name | City  District | Archaeological intervention Code | Chronology by century (C.E.) | Period | References of the archaeological intervention report | Total of Adult Individuals | Total of Qualified Individuals for this study |
| --- | --- | --- | --- | --- | --- | --- | --- | --- |
| 14 | Avinguda Francesc Cambó | Ciutat Vella | 018/01 | 4th to 5th | Late antiquity | (Bordas and Torres, 2006) | 30 | 10 |
|  |  |  | 057/05 | 3rd to 7th |  | (Salazar, 2009) | 3 | 1 |
|  |  |  | 072/84 | 4th to 7th |  | (Granados, 1989) | 8 | 1 |
|  |  |  | 072/86 | 4th to 7th |  | (Granados, 1989) | 51 | 16 |
|  |  |  | 101/03 | 4th to 8th |  | (Medina, 2005) | 2 | 1 |
|  |  |  | 311/04 | 4th |  | (Pastor and Caballé, 2005) | 5 | 1 |
| 15 | Carrer Beates | Ciutat Vella | 162/06; 201/05 | 4th to 7th | Late antiquity | (Arias del Real and Calpena, 2008) | 35 | 3 |
| 16 | Carrer Hort de la Bomba | Ciutat Vella | 096/98; 105/00 | 3rd to 6th | Late antiquity | (Artigues, 2001) | 20 | 7 |
| 17 | Carrer Rec 1-53, 2-356 | Ciutat Vella | 103/08 | 3rd to 4th | Late antiquity | (Castillo, 2009) | 1 | 1 |
| 18 | Carrer Argenteria | Ciutat Vella | 2004/97 | 4th | Late antiquity | (Miró, 1997) | 18 | 6 |
| 19 | Carrer Sant Pau - Carretes | Ciutat Vella | 005/01 | 5th to 8th | Late antiquity | (Calpena, 2001) | 4 | 2 |
| 20 | Avinguda Vilanova 3-11 | Eixample | 137/08 | 9th to 12th | Medieval | (Aguelo et al., 2010) | 43 | 6 |
| 21 | Carrer Comerç 7 | Ciutat Vella | 2032/97 | 9th to 10th | Medieval | (Vila, 1999) | 10 | 7 |
| 22 | Passeig Pujades 1 | Ciutat Vella | 1063/95 | 9th to 10th | Medieval | (Martín, 1997) | 8 | 5 |
| 23 | Sant Agustí Vell | Ciutat Vella | 327/94 | 14th to 15th | Medieval | (Flores, 2007) | 2 | 2 |
| 24 | Horts de Can Cadena | San Martí | 107/19 | 17th | Modern | (Esqué and Fàbregas, 2021) | 36 | 16 |
| 25 | Carrer Rec Comtal | Ciutat Vella | 079/13 | 15th to 16th | Modern | (Martí, 2015) | 10 | 4 |
| 26 | Ronda Sant Antoni | Ciutat Vella | 051/18 | 14th to 16th | Modern | (Triay et al., 2021) | 38 | 5 |
| 27 | Plaça Sant Cugat | Ciutat Vella | 137/05 | 15th to 18th | Modern | (Salvadó et al., 2008) | 23 | 4 |
| 28 | Plaça Ramon Berenguer el Gran | Ciutat Vella | 009/15 | 15th to 17th | Modern | (Artigues 2020) | 4 | 2 |
| 29 | Carrer Floristes de  la Rambla 1-3, 7 | Ciutat Vella | 128/14 | 15th to 18th | Modern | (Antequera 2016) | 4 | 4 |
|  |  |  | 022/15 |  |  | (Tenza 2016) | 5 | 5 |
| **Total** | | | | | | | **694ᵃ** | **226ᵇ** |

ᵃ Total number of adults analyzed macroscopically for this study.

ᵇ Total number of adult individuals qualified for this study, where sex and age could be determined, and at least one of the second metacarpals was used for analysis.

**References to the different reports of the archaeological excavations cited in Supplementary Data Table 1:**

Aguelo. J., Juárez. T., Subiranas, C., 2010. "Arc de Triomf", Anuari d'arqueologia i patrimoni de Barcelona 2009. MUHBA, Institut de Cultura, Ajuntament de Barcelona. p.52-55.

Antequera. F., 2016. Memòria de la intervenció arqueològica al carrer de les Floristes de la Rambla, 7. X/ Jardins del Doctor Fleming. Districte de Ciutat Vella (Barcelona, Barcelonès). Generalitat de Catalunya, Barcelona.

Arias del Real, L., Calpena, D., 2008. Memòria conjunta de la intervenció arqueològica al carrer de les Beates 2-5. Generalitat de Catalunya, Barcelona.

Artigues, P., 2001. Memòria de la intervenció arqueològica al C/ST. Pau Núm. 116 Cantonada Reina Amalia i C/Carretes Barcelona (Barcelona). Generalitat de Catalunya, Barcelona.

Artigues, P., 2020. Memòria de la intervenció arqueològica a la plaça de Ramon Berenguer el Gran i carrer Tapineria. Barcelona (Barcelonès). Febre 2015-setembre 2016. Codis: 009/15-024/15. Generalitat de Catalunya, Barcelona.

Balaguer, N., 2016. Intervenció arqueoantropològica a la necròpoli del Triangle Ferroviari, al jaciment neolític i romà davant de l’Estació de Sant Andreu Comtal, i al jaciment neolític del C/11 de setembre C/Josep Soldevila (Barcelona, Barcelonès). Generalitat de Catalunya, Barcelona.

Bordas, A., Subiranas, C., 2006. Intervenció arqueològica a la prolongació de l’avinguda Francesc Cambó, entre els carrers Giralt el Pellisser Gombau i de Fonollar. Generalitat de Catalunya, Barcelona.

Bordas, A., Torres, G., 2006. Memòria conjunta de las intervencions arqueològiques a: ampliació de l’avinguda Cambó. Ciutat Vella, Barcelona. Generalitat de Catalunya, Barcelona.

Busquets, F., Pastor, I., Beltrán de Heredia, J., Gamarra, A., Moreno, I., 2006. Memòria de la intervenció arqueològica preventiva realitzada a la Plaça Vila de Madrid. Barcelona (Ciutat Vella). Generalitat de Catalunya, Barcelona.

Calpena, D., 2001. Memòria de la intervenció arqueològica preventiva als carrers Sant Pau, Reina Amàlia, Hort de la Bomba i Carretes (Ciutat Vella, Barcelona). Generalitat de Catalunya, Barcelona.

Camarasa, V., Piera , J., 2005. Memòria de la intervenció arqueològica de la zona B, del “projecte d’infraestructures troncal des del carrer Carders a la central de RSU, en el mercat de Santa Caterina, al districte de Ciutat Vella, de Barcelona”. 137/02. Generalitat de Catalunya, Barcelona.

Castillo, R., 2009. Memòria científica de la intervenció arqueològica al Carrer Rec, 1- 53, 2 - 36. Generalitat de Catalunya, Barcelona.

Esqué, M., Fàbregas, M., 2021. Memòria de la intervenció arqueològica preventiva (control arqueològic i excavació arqueològica) al carrer de Menorca, 25 (Horts de Can Cadena); districte de Sant Martí (Barcelona, Barcelonès). Generalitat de Catalunya, Barcelona.

Farré, R., Fernández, A., 1994. Memòria de la intervenció Plaça Comercial Barcelona-Barcelonès. Codi-303. Servei d’Arqueologia del Departament de Cultura de la Generalitat de Catalunya.

Flores, C., 2007. Memòria de la intervenció arqueològica realitzada a la finca núm. 16 de la Plaça de l ’Acadèmia de Barcelona (022/07). Servei d’Arqueologia del Departament de Cultura de la Generalitat de Catalunya.

Granados, J.O., 1989. Sondeig arqueològic a la Avinguda Cambó, (Barcelona). Ajuntament de Barcelona. Servei d’Arqueologia de la Ciutat. Generalitat de Catalunya, Barcelona.

Granados, O., Blasco, M., 1990. Prospeccions arqueològiques al solar de la Caserna de la Guardia civil. Carrer de Sant Pau, Núm. 92 (Ciutat Vella, Barcelona). Generalitat de Catalunya, Barcelona.

Hinojo, E., 2020. Memòria de la intervenció arqueològica preventiva realitzada al mercat de Sant Antoni (carrers del Comte d’Urgell, 1 bis / de Manso, 55-57 / del Comte Borrell, 56 bis i de Tamarit, 154 bis). Districte de l’Eixample -Barcelona (Barcelonès). Generalitat de Catalunya, Barcelona.

Huertas, J., Aguelo, J., 2006. Memòria de la intervenció arqueològica al solar del al Mercat de Santa Caterina. Barcelona (Barcelonès). Generalitat de Catalunya, Barcelona.

Martí, A., 2015. “Carrer del rec Comtal, 17-19. Passatge de Sant Benet, 12”, Anuari d’arqueologia i patrimoni de Barcelona 2014, Institut de Cultura, Ajuntament de Barcelona.

Martín, A., 1997. Memòria de la intervenció arqueològica al Passeig Pujades núm 1, Ciutat Vella – Barcelona. Servei d’Arqueologia del Departament de Cultura de la Generalitat de Catalunya.

Medina, E., 2005. Memòria de la intervenció arqueològica a la prolongació de L’avinguda Francesc Cambó – Porta Cambó, Barcelona. Generalitat de Catalunya, Barcelona.

Miquel, J., 2010. Memòria de la intervenció arqueològica preventiva al ras de planta Baixa de l’església de Santa María del Pi de Barcelona. Generalitat de Catalunya, Barcelona.

Miró, N., 1997. Memòria de la intervenció realitzada als carrers de L’Argenteria i Manresa de Barcelona (Barcelonès). Generalitat de Catalunya, Barcelona.

Moreno, I., Esqué, M., 2013. Memòria de la intervenció arqueològica a l’avinguda del paral·lel, 2-32/Plaça de les Drassanes, 1 / Passeig de Josep Carner, 26-30. Generalitat de Catalunya, Barcelona.

Pastor, I., Caballé, G. 2005. Memòria de la intervenció arqueològica subsidiària de la remodelació de l’Avinguda Francesc Cambó, de Barcelona (Barcelonès). Generalitat de Catalunya, Barcelona.

Prida, D., 2009. Memòria arqueològica de la intervenció efectuada al CA1 de Barcelona Generalitat de Catalunya, Barcelona.

Ravotto, A., 2011. "Drassanes Reials", Anuari d'arqueologia i patrimoni de Barcelona 2010. MUHBA, Institut de Cultura, Ajuntament de Barcelona. p. 34-36.

Salazar, N., 2009. Carrers Álvarez de Castro 1-7 i 2-10, Jaume Giralt 19-43, Gombau 1-11 i 2-16, Mestres Casals i Martorell 1-27 i 2-26, Arc de Sant Cristòfor 1-23 i Fonollar 21-29. Ciutat Vella. Barcelona, Barcelonès. Memòria de la intervenció arqueològica preventiva. Codi MHCB: 057/05. Servei d’Arqueologia del Departament de Cultura de la Generalitat de Catalunya.

Salvadó, I., Vilardell, A., Gallego, J., García, J., 2008. Intervenció arqueològica al Jaciment de la plaça Sant Cugat del Rec, del c/Fonollar, núm. 1-17 i 2-18, del c/Sant Domènec de Santa Caterina, Núm. 1 i 4-6 del c/Forn de la Fonda, núm. 1-5 i 2, del c/Tarròs, núm. 13-17 i 2-22, C/Giralt d’en Pellicer núm. 2-8 (Barcelona, Barcelonès). Generalitat de Catalunya, Barcelona.

Tenza, A., 2016. Memòria de la intervenció arqueològica al carrer de les Floristes de la rambla 1-3. Districte de Ciutat Vella, Barcelona (Barcelonès). Generalitat de Catalunya, Barcelona.

Triay, V., 2006. Memòria arqueològica de la intervenció efectuada al carrer Ample, 5 de Barcelona (El Barcelonès). 2004. Generalitat de Catalunya, Barcelona.

Triay, V., Ramos, J., Medina, E., 2021. Memòria de la intervenció arqueològica preventiva efectuada a la ronda de Sant Antoni, 84 / Carrer de Joaquín Costa, 63 Districte De Ciutat Vella, Barcelona. Codi Servei d’Arqueologia de l’Ajuntament de Barcelona: 051/18. Servei d’Arqueologia del Departament de Cultura de la Generalitat de Catalunya.

Velasco, A., Terrats, N., Gómez, A., Molist, M., 2017. L'assentament de la plaça de la Gardunya a inicis del II mil·lenni: noves dades sobre les ocupacions de l'edat de Bronze inicial al Pla de Barcelona. Quaderns d'Arqueologia i Història de la Ciutat de Barcelona. QUARHIS, 13, p. 70-89.

Vila, J.M., 1999. Memòria de la intervenció arqueològica realitzada al carrer Comerç núm. 7 (intervenció 2032). Barcelona, Barcelonès. Servei d’Arqueologia del Departament de Cultura de la Generalitat de Catalunya.

Vilardell, A., 2009. Memòria de la Intervenció arqueològica al Pou de la Figuera, 2006-2007. Ajuntament de Barcelona. Servei d’Arqueologia de la Ciutat.

**Supplementary data table 2.** Mean bone measurements (in mm) for the entire sample according to age categories, including ANOVA results and corresponding post hoc Tukey test values.

| Age groups | **Measurements** | | | | | |
| --- | --- | --- | --- | --- | --- | --- |
|  | Total length (TL) | SD | Diaphysis total width (DTW) | SD | Medullary Width (MW) | SD |
| Young n=73 | 67.22 | 0.05 | 8.02 | 0.10 | 3.67 | 0.11 |
| Middle n=127 | 68.73 | 0.04 | 8.38 | 0.80 | 3.95 | 0.90 |
| Mature n=26 | 68.34 | 0.08 | 8.49 | 0.16 | 4.10 | 0.22 |
| ANOVA | p =0.057 | | **p =0.011*** | | p =0.084 | |
| Tukey HSD | N.S | | **Young vs. Middle**  **Young vs. Mature** | | N.S | |

**Supplementary data table 3.** Mean bone measurements (in mm) by age groups, divided by sex, with Kruskal–Wallis test values

| Sex | Age groups | **Measurements** | | | | | |
| --- | --- | --- | --- | --- | --- | --- | --- |
|  |  | Total length (TL) | SD | Diaphysis total width (DTW) | SD | Medullary Width (MW) | SD |
| Males | Young (n=29) | 68.75 | 0.33 | 8.52 | 0.08 | 3.99 | 0.07 |
|  | Middle (n=73) | 70.46 | 0.43 | 8.73 | 0.090 | 4.14 | 0.11 |
|  | Mature (n=15) | 69.55 | 0.44 | 8.77 | 0.08 | 4.35 | 0.11 |
|  | Kruskal–Wallis | p =0.156 | | p =0.498 | | p =0.533 | |
| Females | Young (n=44) | 66.21 | 0.41 | 7.69 | 0.07 | 3.46 | 0.10 |
|  | Middle (n=54) | 66.40 | 0.36 | 7.90 | 0.07 | 3.69 | 0.08 |
|  | Mature (n=11) | 66.70 | 0.36 | 8.11 | 0.07 | 3.75 | 0.11 |
|  | Kruskal–Wallis | p =0.923 | | p =0.144 | | p =0.409 | |

**Supplementary data table 4.** Mean bone measurements (in mm) and metacarpal cortical index (in cm), divided by sex, age, and period groups, along with Kruskal–Wallis statistical test results.

| Measurements | Sex | Historical Periods | Young adults | | | | Middle Adults | | | | Mature adults | | | |
| --- | --- | --- | --- | --- | --- | --- | --- | --- | --- | --- | --- | --- | --- | --- |
|  |  |  | *n* | Mean | SD | Kruskal–Wallis | *n* | Mean | SD | Kruskal–Wallis | *n* | Mean | SD | Kruskal–Wallis |
| TL  (Total length) | Males | Roman | 6 | 69.34 | 0.18 | p= 0.763 | 14 | 70.82 | 0.36 | p= 0.345 | 3 | 71.26 | 0.46 | p= 0.284 |
|  |  | Late antiquity | 14 | 68.42 | 0.40 |  | 32 | 68.53 | 0042 |  | 9 | 67.96 | 0.46 |  |
|  |  | Medieval | 4 | 69.75 | 0.27 |  | 15 | 71.53 | 0.55 |  | 1 | 72.70 | - |  |
|  |  | Modern | 5 | 68.18 | 0.39 |  | 12 | 69.68 | 0.44 |  | 2 | 72.54 | 0.03 |  |
|  | Females | Roman | 12 | 64.20 | 0.34 | p= 0.159 | 11 | 64.75 | 0.32 | p= 0.407 | 2 | 64.03 | 0.36 | p= 0.391 |
|  |  | Late antiquity | 18 | 66.50 | 0.42 |  | 24 | 66.53 | 0.37 |  | 7 | 67.11 | 0.41 |  |
|  |  | Medieval | 8 | 68.96 | 0.43 |  | 5 | 67.26 | 0.37 |  | - | - | - |  |
|  |  | Modern | 6 | 65.69 | 0.33 |  | 14 | 67.15 | 0.36 |  | 2 | 67.93 | 0.04 |  |
| DTW  (Diaphysis Total Width) | Males | Roman | 6 | 8.89 | 0.09 | p= 0.642 | 14 | 9.01 | 0.10 | p= 0.096 | 3 | 8.33 | 0.07 | p= 0.327 |
|  |  | Late antiquity | 14 | 8.33 | 0.08 |  | 32 | 8.29 | 0.10 |  | 9 | 8.59 | 0.06 |  |
|  |  | Medieval | 4 | 8.51 | 0.06 |  | 15 | 8.79 | 0.07 |  | 1 | 10.78 | - |  |
|  |  | Modern | 5 | 8.64 | 0.09 |  | 12 | 8.65 | 0.08 |  | 2 | 9.22 | 0.07 |  |
|  | Females | Roman | 12 | 7.60 | 0.05 | p= 0.701 | 11 | 7.81 | 0.07 | p= 0.259 | 2 | 8.04 | 0.06 | p= 0.281 |
|  |  | Late antiquity | 18 | 7.81 | 0.08 |  | 24 | 7.82 | 0.08 |  | 7 | 8.85 | 0.10 |  |
|  |  | Medieval | 8 | 7.48 | 0.06 |  | 5 | 7.58 | 0.06 |  | - | - | - |  |
|  |  | Modern | 6 | 7.84 | 0.07 |  | 14 | 8.20 | 0.05 |  | 2 | 7.63 | 0.00 |  |
| MW  (Medullary Width) | Males | Roman | 6 | 4.06 | 0.07 | p= 0.791 | 14 | 4.77 | 0.13 | p= 0.197 | 3 | 5.05 | 0.06 | p= 0.391 |
|  |  | Late antiquity | 14 | 4.05 | 0.07 |  | 32 | 3.83 | 0.11 |  | 9 | 3.87 | 0.12 |  |
|  |  | Medieval | 4 | 4.17 | 0.04 |  | 15 | 3.91 | 0.07 |  | 1 | 5.22 | - |  |
|  |  | Modern | 5 | 3.60 | 0.11 |  | 12 | 4.04 | 0.09 |  | 2 | 5.05 | 0.03 |  |
|  | Females | Roman | 12 | 3.54 | 0.09 | p= 0.074 | 11 | 3.89 | 0.09 | p= 0.381 | 2 | 4.18 | 0.16 | p= 0.956 |
|  |  | Late antiquity | 18 | 3.56 | 0.10 |  | 24 | 3.51 | 0.08 |  | 7 | 3.70 | 0.11 |  |
|  |  | Medieval | 8 | 2.75 | 0.07 |  | 5 | 3.41 | 0.04 |  | - | - | - |  |
|  |  | Modern | 6 | 3.99 | 0.11 |  | 14 | 3.94 | 0.09 |  | 2 | 3.53 | 0.12 |  |
